# Distributed Smart Device for Monitoring, Control and Management of Electric Loads in Domotic Environments

**DOI:** 10.3390/s120505212

**Published:** 2012-04-26

**Authors:** Ricardo Morales, Francisco J. Badesa, Nicolas García-Aracil, Carlos Perez-Vidal, Jose María Sabater

**Affiliations:** Department of Control and Systems Engineering, Miguel Hernandez University, Avda. de la Universidad S/N, 03202, Elche, Spain; E-Mails: rmorales@umh.es (R.M.); fbadesa@umh.es (F.J.B.); nicolas.garcia@umh.es (N.G.-A.); j.sabater@umh.es (J.M.S.)

**Keywords:** smart device, electrical efficiency, distributed control

## Abstract

This paper presents a microdevice for monitoring, control and management of electric loads at home. The key idea is to compact the electronic design as much as possible in order to install it inside a Schuko socket. Moreover, the electronic Schuko socket (electronic microdevice + Schuko socket) has the feature of communicating with a central unit and with other microdevices over the existing powerlines. Using the existing power lines, the proposed device can be installed in new buildings or in old ones. The main use of this device is to monitor, control and manage electric loads to save energy and prevent accidents produced by different kind of devices (e.g., iron) used in domestic tasks. The developed smart device is based on a single phase multifunction energy meter manufactured by Analog Devices (ADE7753) to measure the consumption of electrical energy and then to transmit it using a serial interface. To provide current measurement information to the ADE7753, an ultra flat SMD open loop integrated circuit current transducer based on the Hall effect principle manufactured by Lem (FHS-40P/SP600) has been used. Moreover, each smart device has a PL-3120 smart transceiver manufactured by LonWorks to execute the user's program, to communicate with the ADE7753 via serial interface and to transmit information to the central unit via powerline communication. Experimental results show the exactitude of the measurements made using the developed smart device.

## 1. Introduction

Construction represents a strategically important sector for the European Union (EU), providing building and infrastructure on which many sectors of the economy depend. The sector provides constructed assets representing 49.6% of the EU Gross Fixed Capital Formation (GFCF). In 2007 the lead market initiative for Europe was launched. Six markets were considered as the targets of this initiative: eHealth, protective textiles, sustainable construction, recycling, bio-based products and renewable energies. According with the EU, sustainable construction can be defined as a new and sustainable paradigm of developers with new solutions. Investors, the construction industry, professional services, industry suppliers and other relevant parties are facing the challenge towards achieving sustainable development, taking into consideration environmental, socio-economic and cultural issues.

This new paradigm embraces a design and management of buildings and constructed assets, drastic improved energy efficiency of buildings, choice of materials, improved building performance as well as interaction with urban and economic development and management. The market for construction is often classified into residential, non-residential, and infrastructure [1]. One of the trends for the residential market reported by “Accelerating the Development of the Sustainable Construction Market in Europe” is the introduction of building management systems jointly with information and communication technologies, which would enable occupants to control a greater variety of functions to have better comfort, save energy and facilitate remote supervision and control of appliances, equipment and security systems.

**Figure 1.**
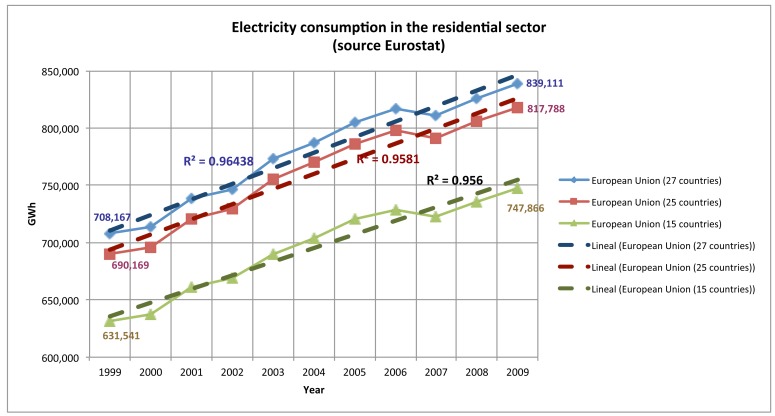
Quantity of electricity consumed by households. Household consumption covers all use of electricity for space and water heating and all electrical appliances. Source: Eurostat.

**Figure 2.**
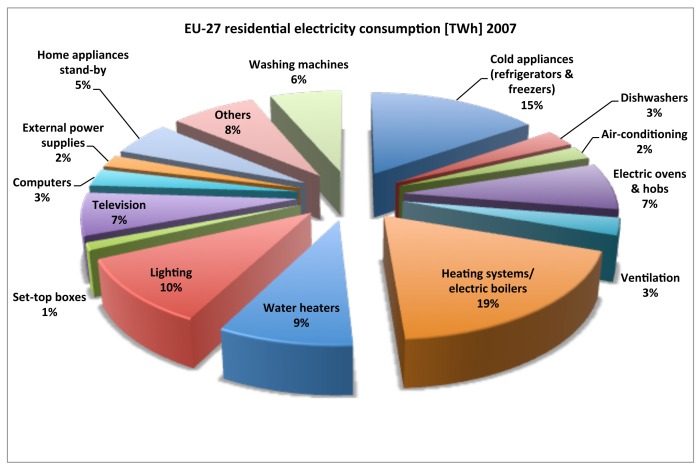
Breakdown of residential electricity consumption in EU-27 in 2007 (source European Commission-Joint Research Center, Institute for Environment and Sustainability).

The residential sector is one of the largest users of energy in the Europe. Electricity consumption of the residential sector for the EU-27 has grown by 18.49% in the period 1999–2009, from 708.176 GWh in 1999 to 839.111 GWh in 2009 and by 3.53% in the period 2007–2009 (Figure 1). The largest electricity consumers in EU-27 households are electric heating systems (18.8%), cold appliances (15.3%), lighting (10.8%) and water heating systems (8.6%) (Figure 2) [2].

Increasing electricity demand is due to many different factors, including:

- More penetration of traditional appliances (e.g., dishwashers, tumble driers, air-conditioners, personal computers, DVD players, broadband equipment, cordless telephones, etc.), many with standby losses.
- Increased use of traditional equipment: more hours of TV watching, more hours of use of personal computer (driven by some tele-working and increased use of internet), more hours of use of washing machines and of hot water.
- Increased number of double or triple appliances, mainly TVs and refrigerators/freezers.
- More single family houses, each with some basic appliances, and larger houses and apartments. This results in more lighting, more heating and cooling systems, and last but not least, older population demanding higher indoor temperatures and all-day heating in winter and cooling in summer, and spending more time at home.

The ICTs are becoming more and more important in terms of electricity consumption, accounting in 2007 for up to 13% in the residential sector (+2% compared to 2004). This is due to a wider penetration of some technologies (*i.e.*, computers, set-top-boxes, modems, external power supplies) as well as a strong penetration of new technologies due to the market transformation (*i.e.*, digital television, larger screens LCD and plasma display television and broadband communication). The stand-by consumption accounts for 5.9% of electricity consumption in EU homes almost the same as computers and dishwashers combined. The reduction of this kind of consumption is the subject of an EU eco-design Regulation and it will be an important driver for achieving energy savings by 2020 (source European Commission-Joint Research Center, Institute for Environment and Sustainability).

Energy efficiency in buildings is currently following two different directions: Passive housing and Intelligent housing. The former refers to simple robust systems that allow exceptionally low energy consumption by integration of available efficient solutions. The later requires an ICT platform that integrates high-tech solutions for energy efficiency. These two directions present different innovation developments but they both focus on enhancing building insulation, indoor climate control systems and energy recovery, and building automation systems. Building automation aims at the integrated optimization of control and performance of building sub-systems such as ventilation, heating and cooling, lightning and electronic equipment. This integration of sub-systems allows buildings to adapt automatically to changing needs and conditions in response to internal or external conditions. The response to changes in conditions is attained through sensing and monitoring technologies. The device presented in this paper can be considered as a building sub-system to monitor, control and management of electric loads in home and building environments.

The term smart sensor was first used in the mid of 1980s. Smart sensor is defined by the Institute of Electrical and Electronics Engineers (IEEE) in the IEEE 1451.2 standard as a sensor that provides functions beyond those necessary for generating a correct representation of a sensed or controlled quantity. This function typically simplifies the integration of the transducer into applications in a networked environment. Moreover, a smart sensor should include certain functionalities such as processing, communication and integration according to the classification given by [3]. The concept of smart sensor is extended to a higher level when the presence of digital microcontrollers or processors of the signal, as a processing subsystem, is taken into account. From a practical point of view, it is possible to have several of these subsystems sharing the same package, so that various types of smart sensors can be considered. The smart sensors are emerging as a promising technology in a large number of application domains: to estimate real-time high-resolution frequency in power systems [4]; to monitor real-time electricity consumption with long-distance communication capabilities [5]; to estimate motion dynamics, inclination and vibration parameters on industrial manipulator robot links based on two primary sensors: an encoder and a triaxial accelerometer [6]; to measure the plant transpiration fusing five primary sensors (air temperature, leaf temperature, air relative humidity, plant out relative humidity and ambient light) [7]. The device presented in this paper can be classified as smart sensor with actuation capability since electrical loads can be switched on/off by the devices according with the user's program and/or the command sent by the central control unit. Take into account this control capability, the device is defined as “smart device”.

From the workplace to the home, different types of end users make use of different kinds of devices/instruments every day. The need to access information concerning these devices/instruments has forced technology to evolve dramatically [8]. As part of the evolution, effective performance monitoring and remote control of these devices are now a primary interest for many commercial and industrial businesses. Due to the advances in electronics and communication networks, the ability to acquire information and even to control devices at fingertips over the existing powerlines is becoming desirable to the general public as well as professionals.

This paper presents the design and development of a smart device for monitoring, control and management of electrical loads at home. The paper is structured in two sections: the first one describe in detail the energy meter of the smart node and the communication between the energy meter-on board processor and between the smart sensor and the central unit; and the second one shows the experimental results obtained using the smart device prototype.

Moreover, this device could be used not only at home. It could be very effective in the industry applying the same idea (switching ON/OFF loads following a specific criteria) or even controlling devices (control theory application) where a bigger bandwidth is needed [9].

## 2. System Overview

The proposed system structure offers the building/home monitoring, control and management of electrical loads based on communications over existing powerlines (no new wires are needed) [5]. The system could be divided into the smart nodes and the central unit. The smart node could monitor the parameters of current, voltage and energy in order to communicate the real time consumption of the electrical load to the central unit. The central unit would analyze the information jointly with the rules provided by the user to take actions like switch off the load and/or sending information messages about overload to the user. The next sections describe in detail the energy meter of the smart node and the communication between the energy meter-on board processor and between the smart sensor and the central unit.

### 2.1. Energy Meter

Big manufacturing companies of high-performance semiconductors have recently designed a series of integrated circuits of increasing complexity and better capacity, specifically to measure the energy transferred to a load connected to an AC line. These circuits (energy monitors) are mixed processors (digital/analog) which provide information of the energy used (active, reactive and apparent), and then transmit it using output pulses of variable frequency or standard serial protocol. Most of the circuits have two inputs, one is proportional to the voltage of the load and the other one is proportional to the current that circulates through the load. The basic functioning consists in digitalizing the signals which are related to the voltage and current in the load and multiplying them, so that the result is proportional to the power in the load [10].

In this paper, we have selected the ADE7753 device, manufactured by Analog Devices. The energy meter has two fully differential voltage input channels. The maximum differential input voltage for input pairs V1P/V1N and V2P/V2N is 0.5 V. However, each analog input channel (V1P/V1N and V2P/V2N) has a Programmable Gain Amplifier (PGA) with possible gain selections of 1, 2, 4 8, and 16 writing respectively bits 0 to 2 and bits 5 to 7 of the gain register. However, by using Bits 3 and 4 in the gain register, the maximum ADC input voltage can be set to 0.5 V, 0.25 V, or 0.125 V. [11]. In this case, authors configured the gain register for a maximum ADC input voltage of 0.5 V in each channel.

### 2.2. Current Measurement

The four most common sensor technologies today for current measurement are: low resistance current shunt, Current Transformer (CT), the Hall effect sensor and Rogowski coil. Low resistance current shunt offers good accuracy at low cost and the current measurement is simple. A good and recent review about current sensing techniques can be consulted in [12]. Ziegler *et al.* summarized in two tables: (i) the performance of the different current sensing techniques using seven features (Bandwidth, DC Capable, Accuracy, Thermal Drift, Isolated, Range and Power Loss) and (ii) the cost and common applications.

All the four sensor technologies are compatible with the selected energy meter IC (ADE7753). The design requirements are: (i) the current measurement technology should be as small as possible; (ii) the ADE maximum differential voltage input channels are 0.5 V; (iii) Current measurement capability up to 16 A. Rogowski coil, combining with digital integrator, offers a cost competitive current sensing technology and could be the best option between the four sensor technologies, but the main drawback is the size since our designed solution should fit inside a Schuko socket. Resistance current shunt technology is a good option taking into account the cost, but we tested it in the lab and the generated heat was too high for this application. CT might be a feasible option too but it has saturation problems. The solution to the saturation problem is to use Mu-metal core CT, but Mu-metal core CTs requires multiple calibration points for both current level and temperature variations. Finally, authors decided to skip this sensor technology. The selected sensor technology was Hall effect sensors. Two Hall effect sensors were tested in the lab: FHS-40P/SP600 manufactured by Lem and ACS712 manufactured by Allegro. The performance of both sensors were satisfactory but finally Lem's solution was selected because of the cost.

**Figure 3.**
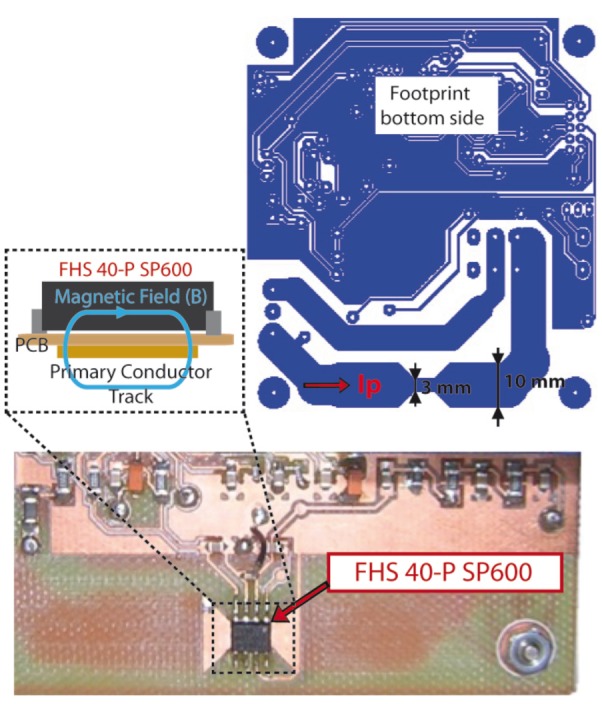
Principles of current measurement using FHS-40P/SP600 current transducer.

FHS-40P/SP600 is an ultra flat SMD open loop integrated circuit current transducer based on the Hall effect principle. It senses the magnetic field generated by the measured current and transforms it into an output voltage. A current flowing in a long thin conductor generates a flux density around it (Figure 3).

### 2.3. Current Signal Conditioning

**Figure 4.**
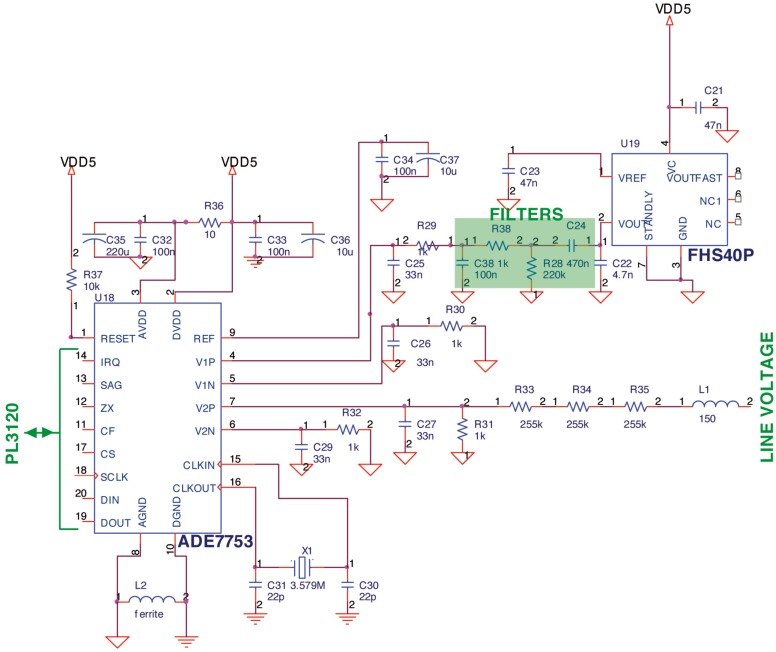
Schematic Detail of ADE7753 and FHS-40P connection.

As it was commented previously, FHS-40P/SP600 output voltage must be conditioned in order to be acquired by the ADE7753 circuit. To do that, the DC component of the current sensor should be removed since the ADE maximum differential voltage input channel is ±0.5 V. The DC component can be eliminated using a basic 0 Hz high pass filter. The main drawback of this solution is that the filter produces a delay in the signal which affects the correct performance of the ADE7753. Therefore, a low pass filter was designed to counteract the effect of signal delay produced by the high pass filter. The design of the two filters must fulfill two main objectives: (i) to remove DC component and; (ii) to nullify the total delay produced by the two filters. Taking into account this requirements, a RC high pass filter was calculated: *C*_24_ = 470 *nF, R*_28_ = 220 *K*Ω and *θ*_1_ = 1.76 and a RC low pass filter was calculated too: *C*_38_ = 100 *nF, R*_38_ = 1 *K*Ω and *θ*_2_ = −1.79 (Figure 4).

### 2.4. Voltage Measurement

A resistive voltage divider has been designed to attenuate the line voltage down to ±0.5 V, because the input voltage channel of the ADE7753 has a a maximum differential voltage of ±0.5 V. The line voltage attenuation is carried out by a simple resistor divider as shown in Figure 4. The topology of the network is such that the phase matching between input channel 1 and input channel 2 is preserved. As can be seen from Figure 4, the 3 dB frequency of this network is determined by *R*_31_ = 1 *K*Ω and *C*_27_ = 33 *nF*. This is due to *R*_33_ – *R*_35_ = 225 *K*Ω being much greater than *R*_31_ = 1 *K*Ω.

### 2.5. Distributed Control System

Powerline has been selected as a physical transport media due to the following advantages: the installed powerline can be used as a communication channel; there are no interferences with other devices (like in the radio communication case); there is the possibility to put the distributed smart modules in every place (in a electrical outlet); there is no need to have an extra power source (usually, in a bus cable domotic system, there is a direct voltage generated by a power supply and distributed on the whole domotic net). The main drawback of the powerline communication is related with the electrical noise generated by home appliances and/or by a near electrical cabinets.

Many, but not all, manufacturers of Home and Building Automation system sell modules that can communicate over the powerline. The main reason to select LonWorks PL-3120 is that it uses a dual-carrier frequency signaling technology to provide superior communication reliability in the face of interfering noise sources. With the dual carrier frequency feature the last two retries of acknowledged service messages are sent using the secondary carrier frequency. Thus, in A-band and when acknowledged service is used with three retries (four total tries), the first two tries are sent using the 86 kHz primary carrier frequency. If the last two tries are needed to complete the transaction, they are sent (and acknowledged) using the 75 kHz secondary carrier frequency. Similarly, for C-band operation the primary and secondary frequencies are 132 kHz and 115 kHz respectively. A minimum of two retries must be used if the PL-3120 is to be able to use both carrier frequency choices.

The core of the our developed system is the LonWorks PL-3120 [13]. The PL-3120 Smart Transceivers integrate a Neuron processor core with an ANSI/EIA-709.2 compliant power line transceiver within a single Integrated Circuit (IC), eliminating the need for an external transceiver. The Neuron processor core is composed of three processors. These processors are assigned to the following functions: (i) Processor 1 is the MAC layer processor that handles layers 1 and 2 of the 7-layer LonTalk protocol stack; (ii) Processor 2 is the network processor that implements layers 3 through 6 of the LonTalk protocol stack; (iii) Processor 3 is the application processor. It executes the code written by the user, together with the operating system services called by user code. The primary programming language used by applications is Neuron C, a derivative of the ANSI C language optimized and enhanced for Lonworks distributed control applications.

The PL-3120 provides 12 I/O pins which can be configured to operate in one or more of 38 predefined standard input/output modes. Combining a wide range of I/O models with two on-board timer/counters enables the PL 3120 to interface with application circuits using minimal external logic or software development. The PL-3120 also features a full duplex hardware UART supporting baud rates of up to 115 kbps, and an SPI interface that operates up to 625 kbps. In this application, 8 I/O pins (from IO2 to IO10) have been configured to interchange information with ADE7753.

Serial Peripheral Interface (SPI) interface has been selected to communicate the PL-3120 (configured as Master in the SPI bus) and the ADE7753 (configured as Slave in the SPI bus). In short, the selected configuration mode of PL-3120 is Neurowire master mode in which pin IO8 is the clock (driven by the PL-3120), pin IO9 is always output (serial data output) and IO10 is always input (serial data input) as it can be seen in Figure 4. Moreover, pin IO7 of PL-3120 has been configured as a select pin to enable the ADE7753 serial communication mode.

**Figure 5.**
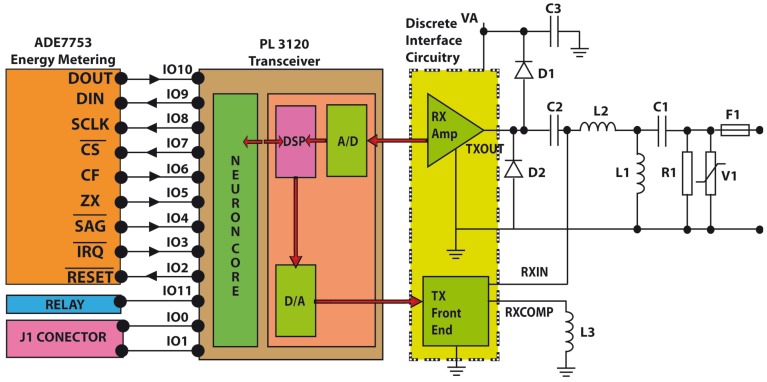
A schematic detail of the PL-3120 and ADE7753 connection.

The other pins has been configured as: (i) pin IO2 has been configured as digital output to reset ADE7753; (ii) pin IO3 has been defined as digital input to control if an previously programmed interruption request has been produced in ADE7753; (iii) pin IO4 is configured as digital input and it is connected to the SAG output of the ADE7753 to monitor the power supply input to ADE7753; (iv) pin IO5 is configured as digital input to monitor the zero-crossing detection (we do not use this signal directly since we detect the zero-crossing through interrupt status register of ADE7753); (v) pin IO6 is configured as digital input and it is connected to the CF output of ADE7753 for calibration purpose; (vi) pin IO11 has been configured as digital output to control the relay (Load switch on/off). A schematic detail of the PL-3120 and ADE7753 connection can be seen in Figure 5.

In communication mode, the ADE7753 is waiting for a write operation to its communication register. The data written to the communication register indicates whether the next serial data communication operation will be a read or a write operation and also which register is accessed. In short, the communication register of ADE7753 is one byte long: bit 7 determines whether the next data transfer operation is a read or a write operation, bit 6 is reserved and bit 5 to 0 are used to address the register of ADE7753 to be accessed. In this design, the PL-3120 configured as Neurowire master mode uses the function *io_in()* and *io_out()* from Neuron C programming language [14]. Neuron C is a programming language based on ANSI C that is designed for Neuron Chips and Smart Transceivers like the PL-3120.

The smart device for monitoring, control and management of electric load can be considered as a Lonworks device since the PL-3120 is used to communicate data from/to other devices and the central unit. To do this, network variables to monitor *I_RMS_, V_RMS_*, instantaneous power, active power, reactive power, active energy, reactive energy, frequency and to control the connection of the electric load are defined within the program that runs on all the individual smart devices.

## 3. Experimental Results

**Figure 6.**
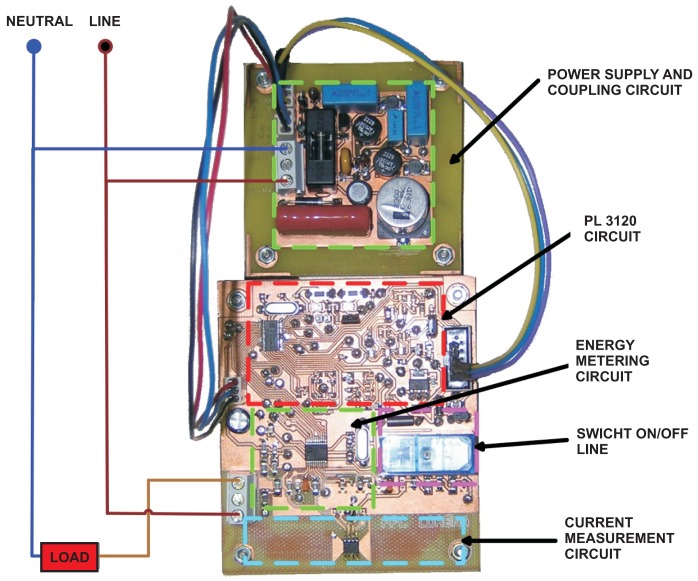
Setup to test the smart device.

To evaluate the electronic prototype performance, a setup with different typical loads was build to test the system (Figure 6). To control and monitor this smart device, a central computer was used keeping in mind that it will be replaced in a near future by a central control unit based on a microcontroller with a Human Machine Interface (HMI) system (Graphic LCD display with touch technologies). The application program to test the functionalities of the smart device reads the following data: *I_RMS_, V_RMS_* and active, reactive and apparent energy and control the relay used to switch on/off the load (Table 1).

The experiment to measure the accuracy of the measurement made by the developed device was carried out using a load with two known energy consumptions (the load is an oil radiator with 2 heat settings I and II) (Table 1) and a CW120 power meter manufactured by Yokogawa. The real measurement of *I_RMS_* is more or less the same as the measurement obtained with the developed smart device. Moreover, the active and apparent energy has been measured with the developed device and with an energy analyzer to compare the measurements and to compute the error (Table 2). The ADE7753 achieves the integration of the active power signal by continuously accumulating it in an internal nonreadable 49-bit energy register [11]. The active energy register (AENERGY[23:0]) represents the upper 24 bits of this internal register. This discrete time accumulation or summation is equivalent to integration in continuous time and the discrete time sample period (T) for the ADE7753 accumulation register is 1.1 *μs* (4/CLKIN where CLKIN in ADE7753 logic input to provide a clock source. The recommended clock frequency is 3.579545 MHz). The relationship between watt-hours accumulated and the quantity read from AENERGY can be determined from the amount of active energy accumulated over time with a given load:

(1) $$\frac{\text{Wh}}{\text{LSB}}=\frac{\text{Load}(W)\cdot \text{AccumulationTime}(s)}{\text{LAENERGY}\cdot 3600\frac{s}{h}}$$

where *AccumulationTime* can be determined from the line cycle period, measured by the ADE7753 in the PERIOD register (*Period*), and the number of half line cycles in the accumulation, fixed by the LINECYC register.

(2) $$\text{AccumulationTime}(s)=\frac{\text{Period}\cdot \text{LINECY C}\cdot 8/\text{CLKIN}}{2}$$

The ADE7753 achieves the integration of the apparent power signal in the same way of the active power signal as it was commented above. The apparent energy register (VAENERGY[23:0]) represents the upper 24 bits of this internal register. The relationship between VAh accumulated and the quantity read from *VAENERGY* can be determined from the amount of active energy accumulated over time with a given load:

(3) $$\frac{\text{VAh}}{\text{LSB}}=\frac{\text{Load}(VA)\cdot \text{AccumulationTime}(s)}{\text{LV AENERGY}\cdot 3600\frac{s}{h}}$$

The apparent and active power provided by ADE7753 do not have the same scaling and thus cannot be compared directly one to the other.

## 4. Conclusions

**Figure 7.**
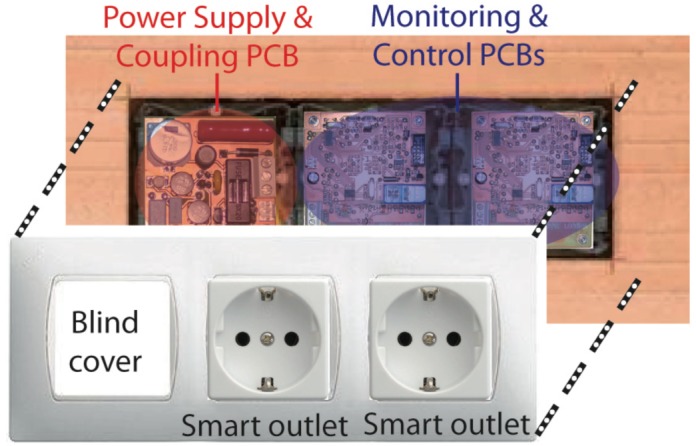
Boards and functional blocks to install the smart sensor inside a Schuko socket.

In this paper, a cost-effective smart device for monitoring, control and management of electric loads is presented. The main features are: (i) Compactness of the design which allows it to be installed inside a Schuko socket; (ii) Communications over existing powerline (no new wires); (iii) Energy metering IC and relay control of the load (switch on/off). The main application of the developed device is saving electrical energy in home environments and preventing accidents produced by different kind of electrical devices (such as iron) used in domestic tasks. The development of intelligent control algorithms to save energy and to prevent accidents is one of the authors' next works. The designed smart sensor have had to be divided into two Printer Circuited Boards (PCBs) due to the size of the components of the coupling circuit. To install the smart device inside a Schuko socket, the lost of one outlet in order to locate the power supply and coupling circuit board for other three or four smart devices has to be assumed. Figure 7 shows PCBs and functional blocks in a typical installation inside a Schuko socket.

## Figures and Tables

**Table 1.** Measurement of *I_RMS_*.

**Type of load**	***I_RMS_* Register ADE7753**	**Measurement ADE7753**	**Real Value**
No Load	0*x*000043	510^−4^ *A*	0 *A*
Load I	0*x*09697*F*	4.465 *A*	4.46–4.47 *A*
Load II	0*x*0*AD*18*A*	5.132 *A*	5.13–5.14 *A*
Load I and II	0*x*140*F*84	9.516 *A*	9.52–9.53 *A*

**Table 2.** Measurement of Energy.

**Measure**	**Active Energy**	**Aparent Energy**
Register ADE7753	0*xCFF*79	0*xB*04*C*
Measurement ADE7753	40.50 *Wh*	40.49 *V Ah*
Real Measurement	40.42 *Wh*	40.42 *V Ah*
Error %	0.20 %	0.16 %
